# Short term results in a population based study indicate advantage for laparoscopic colon cancer surgery versus open

**DOI:** 10.1038/s41598-023-30448-8

**Published:** 2023-03-16

**Authors:** Josefin Petersson, Peter Matthiessen, Kaveh Dehlaghi Jadid, David Bock, Eva Angenete

**Affiliations:** 1 Department of Surgery, SSORG - Scandinavian Surgical Outcomes Research Group, Institute of Clinical Sciences, Sahlgrenska Academy, University of Gothenburg, 416 85 Gothenburg, Sweden; 2 Royal Brisbane and Women’s Hospital, Brisbane, QLD Australia; 3 Department of Surgery, Örebro University Hospital, Örebro, Sweden; 4 Department of Surgery, School of Medicine and Health Sciences, Örebro University, Örebro, Sweden; 5 School of Public Health and Community Medicine, Institute of Medicine, University of Gothenburg, Gothenburg, Sweden; 6 Department of Surgery, Sahlgrenska University Hospital, Region Västra Götaland, Gothenburg, Sweden

**Keywords:** Colon cancer, Colorectal surgery

## Abstract

The aim of this study was to compare LAP with OPEN regarding short-term mortality, morbidity and completeness of the cancer resection for colon cancer in a routine health care setting using population based register data. All 13,683 patients who were diagnosed 2012–2018 and underwent elective surgery for right-sided or sigmoid colon cancer were included from the Swedish Colorectal Cancer Registry and the National Patient Registry. Primary outcome was 30-day mortality. Secondary outcomes were 90-day mortality, length of hospital stay, reoperation, readmission and positive resection margin (R1). Weighted and unweighted multi regression analyses were performed. There were no difference in 30-day mortality: LAP (0.9%) and OPEN (1.3%) (OR 0.89, 95% CI 0.62–1.29, *P* = 0.545). The weighted analyses showed an increased 90-day mortality following OPEN, *P* < 0.001. Re-operations and re-admission were more frequent after OPEN and length of hospital stay was 2.9 days shorter following LAP (*P* < 0.001). R1 resections were significantly more common in the OPEN group in the unweighted and weighted analysis with *P* = 0.004 and *P* < 0.001 respectively. Therefore, the favourable short-term outcomes following elective LAP versus OPEN resection for colon cancer in routine health care indicate an advantage of laparoscopic surgery.

## Introduction

Surgery is the mainstay of treatment for colon cancer and since the introduction of laparoscopic surgery for colon cancer in the early 1990s, the technique has been proven oncologically safe with the advantage of enhanced recovery^1–3^. International studies including randomized controlled trials have demonstrated less blood loss, reduced pain, shorter length of hospital stay along with improved quality of life during the first postoperative month^1,4^. Regardless of the approach, the resection must follow essential oncological principles including central ligation of the primary vessel, proper mesocolic excision, an adequate resection margin and a surgical specimen containing a minimum of 12 lymph nodes^5–9^. It can be technically challenging to perform laparoscopically for T4 tumours although recent meta-analyses and register studies have indicated that LAP is safe and possibly even superior with regard to oncological outcomes when compared to OPEN even for T4 tumours^10–12^.

Furthermore, one fourth of patients presenting with colon cancer have stage IV cancer at time of diagnosis and the role for laparoscopic surgery for this group remains undetermined.

Results from randomized controlled trials and single institutions studies tend to reflect the surgical expertise available at specialized centres. Data on short-term morbidity and mortality from high quality population-based studies reflecting standard care have indicated potential favourable short-term morbidity and mortality after laparoscopic surgery^7,11,13–15^. However, few of these population-based studies have been able to control for elective and emergency surgery or adjust for risk-factors such as comorbidities and BMI.

Despite the encouraging results, the implementation of laparoscopic surgery for colon cancer in Sweden has been slow compared to other laparoscopic procedures, as in many other countries^16–18^. However, since 2010, there has been a steady increase in the use of laparoscopic technique for elective colon cancer in Sweden, surpassing 50% in 2017, still many surgeons perform open resections^19,20^.

The objective of this study was to compare LAP with OPEN in a recent national cohort regarding short-term mortality as well as short-term morbidity and completeness of cancer resection in routine health care for all colon cancers using data from a high quality population based register, including all tumour stages.

## Methods

### Study population and variables

Data was collected from the Swedish Colorectal Cancer Registry (SCRCR) which has a coverage of 99.8% of diagnosed colorectal cancers in Sweden and combined with the National Patient Registry (NPR) for 30 and 90-day morbidity and mortality data. From 1987 onwards all in-patient health care is registered in the NPR. External validation has reported the reliability of the registry to be high^21,22^.

The study adhered to the Declaration of Helsinki. Individual informed consent was waived as this study was observational in nature, ethics approval was obtained from the regional ethics committee Uppsala, Dnr 2018/129 and Dnr 2019/01787^23^.

This nationwide population study included all patients identified in the SCRCR with colon cancer staged I-IV situated in the right colon (including caecum, ascending and hepatic flexure) or the sigmoid colon diagnosed between January 2012 and December 2018 who subsequently underwent elective colonic resection. Robotic laparoscopic surgery was infrequently used for colon cancer surgery, and conventional laparoscopic surgery and robotic laparoscopic surgery were therefore analysed as one group. Robotic laparoscopic surgery has been registered in SCRCR since 2014. Cancers of the transverse and descending colon are less common, are not subjected to a high degree of standardized surgical resection, are generally considered as demanding from a surgical-oncological point of view, and when performed laparoscopically, entail an increased risk for conversion. For these reasons they were not included in the present study^5,24^. Subgroup analyses were performed including Stage I–III disease.

Primary outcome was 30-day mortality. Secondary outcomes were 90-day mortality, re-admissions within 30- and 90-days, re-operations within 30 days and length of hospital stay together with clinical anastomotic leak, positive resection margin (R1) and number of lymph nodes as reported in NPR and SCRCR. Re-admission was defined as at least one re-admission as recorded in the NPR and similarly re-operation was defined as at least one re-operation as recorded in SCRCR. The definition of R1 in the register is microscopic growth of tumor cells in the resection margin and the information is recorded as “yes”, “no”, “uncertain” or “unable to comment”. Patients with resection margin recorded as uncertain, unable to comment or missing (1.1% and 1.8% in the laparoscopic and open group, respectively) were all defined as missing in the analyses. All data is registered and reported electronically to the registry. With regard to anastomotic leakage, the registry has no formal definition, and a checkbox alternative (yes/no) is used.

The variables age, tumour stage (pTNM), BMI, type of surgery and ASA class were defined as potentially and observable confounding variables for short-term mortality and morbidity including length of hospital stay. Year of surgery was also included as a potential confounder since the proportion of laparoscopic surgery increased substantially 2012–2018, over which time period a concurrent overall improvement in outcome was plausible. Completeness of resection was further adjusted for characteristics indicating more advanced tumour including T stage, N stage, level of vascular- and perineural invasion and grade of cancer differentiation. There were no missing values with regard to the primary outcome; for the secondary outcomes missing values were recorded and found to be equally distributed between patients undergoing open or laparoscopic surgery.

### Statistical analysis

A statistical analysis plan was agreed upon prior to analysing the data. All analyses were performed according to intention to treat. Patient characteristics were presented as frequencies and percentages or median (interquartile range, IQR) where appropriate. Group differences were quantified by *p*-values from Chi-square and Mann Whitney U test for categorical and continuous variables, respectively. The effect of surgical technique upon the outcomes was quantified by means of regression models where the identified confounders were adjusted for by including them as factors or covariates. The following variables were adjusted for: age, BMI, ASA class, tumour stage (pTNM), type of surgery and and year of surgery. The analysis of R1 versus R0 were further adjusted for: T stage, N stage, proportion of vascular- and perineural invasion and cancer grade (low or high). Logistic regression was used to analyse binary outcomes (mortality, re-operation, re-admission, R1 versus R0 and ≥ 12 versus < 12 lymph nodes). The reason for this choice of model was that follow up time was short, equal for all patients, without significant concerns of loss to follow up. The continuous outcome, length of stay, was log transformed before linear regression was performed. A second weighted multivariate regression analysis was also performed using the inverse probability treatment weighted (IPTW) method. Patients were weighted based on their propensity scores. Propensity scores were computed from a logistic regression model using the identified confounders previously reported. Results are presented as frequencies, percentages for laparoscopic versus open surgery with 95% confidence interval, *p*-values with odds ratios provided for logistic regression analyses. All analyses were performed using SPSS® version 25 and R^25^.

## Results

### Demographic characteristics

A total of 13,683 patients were registered in the SCRCR as diagnosed with colon cancer stage I–IV between 2012 and 2018 and subsequently underwent colonic resection, right hemicolectomy, sigmoid resection or high anterior resection.

**Figure 1:**
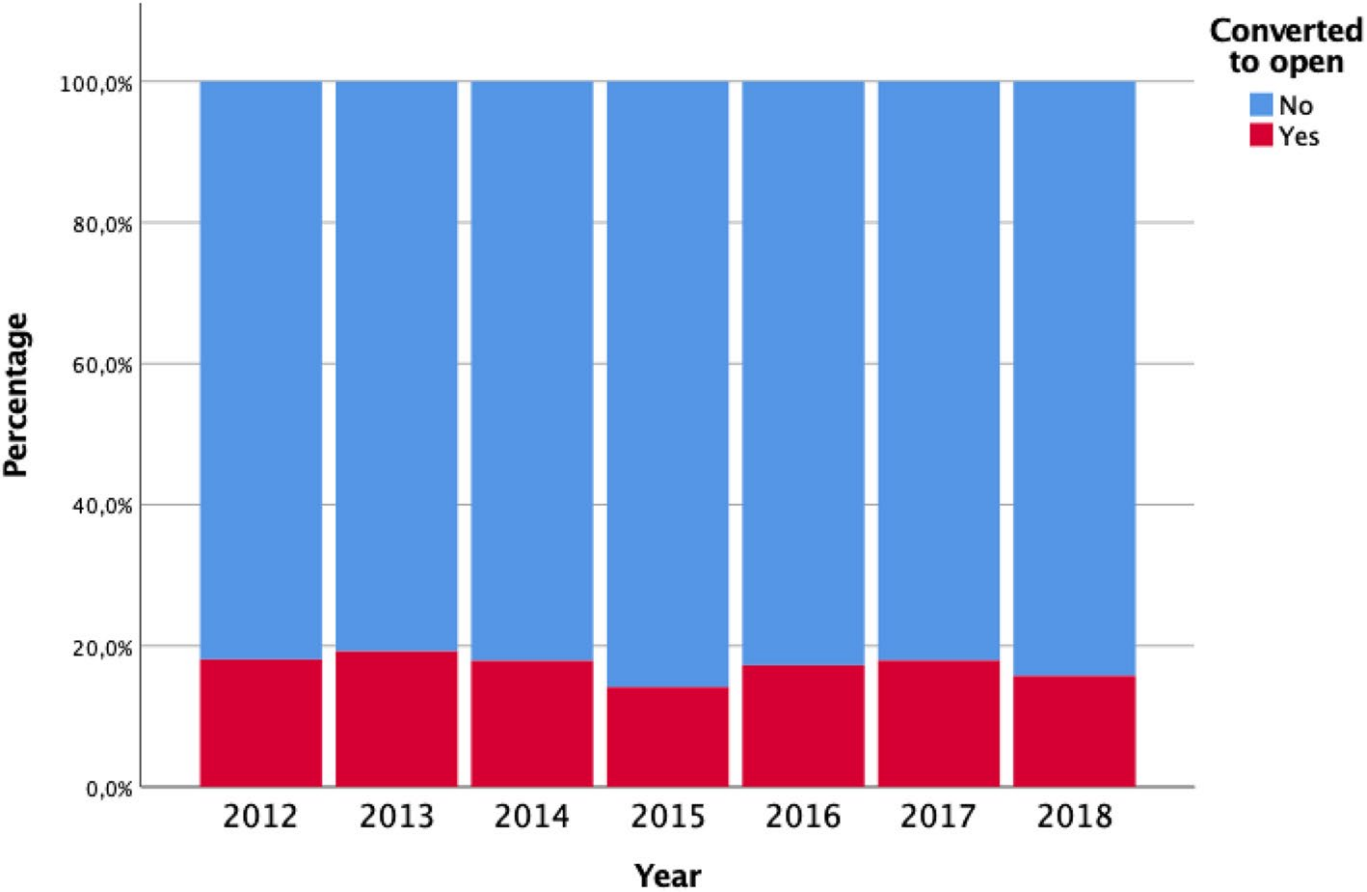
Percentage of laparoscopic procedures converted to open per year.

Among the 13,683 included patients, 38.1% underwent laparoscopic procedures. Laparoscopic procedures increased from 15.4% to 57.2% of all resections, however conversion rate remained unchanged over the study period 18.1% in 2012, 19.9% in 2017 and 15.7% in 2018 (Fig. 1).

Comorbidity measured using American Society of Anaesthesiologists classification (ASA) stage III/IV and male sex was more frequent in the OPEN group compared to the LAP group (Table 1). There were no significant difference with regard to BMI and only a very small difference in age. (Table 1). OPEN surgery was the most common approach for all tumour locations and hepatic flexure tumours were least likely to be performed LAP. Right hemicolectomies were predominantly performed with OPEN technique whereas sigmoid resections were more likely to be performed LAP (Table 2). The LAP technique had statistically significant less bleeding but longer operating time (Table 2). Colonic perforation irrespective of cause was significantly more prevalent in the OPEN group compared to the LAP group 1.4% vs 0.7%, *P* =  < 0.001, of the perforations in the OPEN group, 60,3% were reported as perforations at tumour site (Table 2). The OPEN group displayed more advanced cancers and also more frequently demonstrated potentially negative prognostic tumour characteristics, including higher level of vascular- and perineural invasion and high-grade cancer compared to the LAP group. (Table 2).

**Table 1:** Clinical characteristics.

	Laparoscopic (N = 5208)	Open (N = 8475)	*P*-value
Age in years, median (range)	73 (66–80)	74 (67–81)	0.002
Male, n (%)	2585 (49.6)	4455 (52.6)	0.001
Gender missing, n	9	2	
ASA** I-II/ASA III-IV, (%)	69.1/29.4	62.8/35.8	< 0.001
ASA missing, n	77	120	
BMI***, median (range)	25.7 (23.2–28.6)	25.5 (23.0–28.7)	0.159
BMI missing, n	90	265	
Tumour location, n (%)			< 0.001
Caecum	1491 (28.6)	2462 (29.1)	
Ascendens	1320 (25.4)	2373 (28.0)	
Hepatic flexure	219 (4.2)	891 (10.5)	
Sigmoid	2178 (41.8)	2749 (32.4)	
Tumour location missing, n	0	0	
T stage (cT), n (%)			< 0.001
T1–T2	1695 (32.5)	1530 (18.0)	
T3	2181 (41.9)	3106 (36.6)	
T4	248 (4.8)	1428 (16.8)	
Missing, n	1084	2411	
N stage (cN), n (%)			< 0.001
N0	3084 (59.2)	4121 (48.6)	
N1–N2	1695 (32.5)	3240 (38.2)	
Missing, n	429 (8.2)	1114(13.1)	
cTNM, n (%)			< 0.001
I	1368 (26.3)	1192 (14.1)	
II	1017 (19.5)	1509 (17.8)	
III	1548 (29.7)	2633 (31.1)	
IV	235 (4.5)	852 (10.0)	
Missing, n	1040	2289	

Percentage given as percentage of column unless otherwise noted, **ASA = American Society of Anaesthesiologists classification; ***BMI = body-mass index.

**Table 2:** Intraoperative and pathological characteristics.

	Laparoscopic N = 5208	Open N = 8475	*P*-value
Surgical resection, n (%)			< 0.001
Right hemicolectomy	3030 (58.2)	5726 (67.6)	
Sigmoid resection	1797 (34.5)	2303 (27.2)	
High anterior resection	381 (7.3)	446 (5.3)	
Missing, n	0	0	
Converted to open, n (%)	883 (17.0)	n/a	n/a
Converted to open missing, n	4	n/a	
Operating time, mean (SD)	193.2 (76.8)	184.9 (96.6)	< 0.001
Missing, n	31	96	
Intraoperative bleeding in ml, mean (SD)	87.2 (163.6)	215.6 (384.3)	< 0.001
Intraoperative bleeding in ml missing, n	146	265	
Colonic perforation n (%)	37 (0.7)	116 (1.4)	< 0.001
Missing , n	19	70	
Percentage of perforations close to tumour	27.0	60.3	0.001
Perforation close to tumour missing, n	1	4	
T stage (pT), n (%)			< 0.001
T1	558 (10.7)	466 (5.5)	
T2	1085 (20.8)	1135 (13.4)	
T3	2892 (55.5)	4916 (58.0)	
T4	671 (12.9)	1951 (23.0)	
Missing, n	2	7	
N stage (pN), n (%)			< 0.001
N0	3335 (64.0)	4755 (56.1)	
N1	1314 (25.2)	2227 (26.3)	
N2	558 (10.7)	1492 (17.6)	
Missing, n	1	1	
pTNM, n (%)			< 0.001
I	1347 (25.9)	1292 (15.2)	
II	1927 (37.0)	3247 (38.3)	
III	1718 (33.0)	3082 (36.4)	
IV	216 (4.1)	852 (10.1)	
Missing, n	0	2	
Vascular invasion—yes, n (%)	1351 (26.1)	2607 (30.8)	< 0.001
Missing, n	56	110	
Perineural invasion—yes, n (%)	651 (12.5)	1447 (17.1)	< 0.001
Missing, n	75	204	
Cancer differentiation—high grade, n (%)	927 (17.8)	2180 (25.7)	< 0.001
Missing, n	151	388	

Percentage given as percentage of column.

### Outcomes

#### 30 and 90- day Mortality

The overall 30-day mortality was 1.2%, comprising 113 patients (1,3%) in the OPEN group and 47 patients (0.9%) in the LAP group (OR 0.89, 95% CI 0.62–1.29, *P* = 0.545), (Table 3) The overall 90-day mortality was 313 (2.3%), 234 (2.8%) in patients who had undergone OPEN surgery and 79 (1.5%) in LAP surgery (OR 0.77, 95% CI 0.58- 1.02, *P* = 0.065 in the unweighted analysis) (Table 3). Similar results were found in the subgroup analysis including stage I–III disease however, in the weighted analysis including all cancer stages the increase in 90-day mortality became significant favouring the LAP group (Table 3 and 4).

**Table 3:** Primary and secondary outcomes cTNM I–IV—Unweighted regression analysis and Weighted regression analysis.

	Laparoscopic N = 5208	Open N = 8475	Unadjusted		Unweighted Regression		Weighted Regression	
			OR (95% CI)	*P*-value	OR (95% CI)	*P*-value	OR (95% CI)	*P*-value
30-day mortality	47 (0.9)	113 (1.3)	0.674 (0.479, 0.949)	0.024	0.893 (0.618, 1.289)	0.545	0.951 (0.758, 1.194)	0.667
Missing, n	0	0						
90-day mortality	79 (1.5)	234 (2.8)	0.542 (0.419, 0.702)	< 0.001	0,770 (0.582 , 1.017)	0.065	0.728 (0.612, 0.867)	< 0.001
Missing , n	0	0						
Anastomotic leak	161 (3.1)	299 (3.5)	0.872 (0.718, 1.060)	0.169	0.801 (0.649, 0.989)	0.039	0.794 (0.692, 0.910)	0.001
Re-operation 30-days, n (%)	315 (6.1)	635 (7.5)	0.796 (0.692, 0.915)	0.001	0.846 (0.727, 0.982)	0.030	0.816 (0.772, 0.861	< 0.001
Missing n, (%)	20 (0.4)	24 (0.3)						
Re-admission 30-days	747 (14.3)	1571 (18.5)	0.736 (0.669, 0.809)	< 0.001	0.808 (0.728, 0.896)	< 0.001	0.775 (0.726, 0.828)	< 0.001
Re-admission 90-days	1163 (22.3)	2401 (28.3)	0.727 (0.671, 0.788)	< 0.001	0.822 (0.753, 0.898)	< 0.001	0.754 (0.712, 0.797)	< 0.001
< 12 nodes, n (%)	303 (5.8)	497 (5.9)	0.991 (0.855, 1.148)	0.904	1.086 (0.925, 1.276)	0.314	1.127 (1.015, 1.250)	0.024
Missing, n (%)	30 (0.6)	54 (0.6)						
R1 vs R0			0.462 (0.366, 0,583)	< 0.001	0.681 (0.526, 0.883)	0.004	0.678 (0.579, 0.794)	< 0.001
R1, n (%)	94 (1.8)	322 (3.8)						
R0, n (%)	5055 (97.1)	8001 (94.4)						
Missing, n (%)	59 (1.1)	152 (1.8)						

Percentage given as percentage of column.

**Table 4:** Primary and secondary outcomes cTNM I–III—Logistic regression analysis.

	Laparoscopic N = 4973	Open N = 7623	Unadjusted		Adjusted	
			OR (95% CI)	*P*-value	OR (95% CI)	*P*-value
30-day mortality	44 (0.9)	101 (1.3)	0.665 (0.466, 0.949)	0.025	0.872 (0.596, 1.277)	0.482
Missing, n	0	0				
90-day mortality	75 (1.5)	190 (2.5)	0.599 (0.457, 0.784)	< 0.001	0.814 (0.609, 1.089)	0.166
Missing , n	0	0				
Anastomotic leak 30-days	153 (3.1)	268 (3.5)	0.871 (0.712, 1.066)	0.181	0.796 (0.641, 0.989)	0.040
Re-operation 30-days, n (%)	306 (6.1)	577 (7.6)	0.802 (0.694, 0.925)	0.003	0.862 (0.739, 1.006)	0.060
Missing n, (%)	18 (0.4)	19 (0.2)				
Re-admission 30-days	711 (14.3)	1328 (17.4)	0.791 (0.716, 0.873)	< 0.001	0.842 (0.756, 0.938)	0.002
Re-admission 90-days	1070 (21.5)	2027 (26.6)	0.757 (0.695, 0.824)	< 0.001	0.825 (0.753, 0.905)	< 0.001
< 12 nodes, n (%)	293 (5.9)	436 (5.7)	1.031 (0.885, 1.200)	0.699	1.024 (0.866, 1.212)	0.778
Missing, n (%)	26 (0.5)	50 (0.7)				
R1 vs R0			0.463 (0.360, 0.595)	< 0.001	0.693 (0.524, 0.916)	0.010
R1, n (%)	81 (1.6)	262 (3.4)				
R0, n (%)	4841 (97.3)	7248 (95.1)				
Missing, n (%)	51 (1.0)	113 (1.5)				

Percentage given as percentage of column.

#### Short-term morbidity

A total of 950 patients required one or more re-operations within 30 days. Re-operations were more frequent in the open group compared to the LAP group (Open 7.5% vs LAP 6.0%. This lower prevalence of re-operations in the LAP group was statistically significant both in the unweighted and weighted regression analysis at 30-days postoperatively (Table 3). Re-admissions were also more frequent in the OPEN group compared to the LAP group both at 30- and 90-days postoperatively in the unweighted and weighted regression analysis and in the subgroup analysis including only cTNM I-III (Table 3 and 4). Length of hospital stay was 2.9 days shorter following LAP surgery 6.5 vs 9.4 days with *P* < 0.001 in both the unweighted and weighted analysis (Table 5). Similar results were found in the subgroup analysis including Stage I–III disease (Table 6).

**Table 5:** Primary and secondary outcomes cTNM I–IV—Unweighted regression analysis and Weighted regression analysis.

	Laparoscopic N = 5208	Open N = 8475	Unadjusted		Unweighted regression		Weighted regression	
			OR (95% CI)	*P*-value	OR (95% CI)	P-value	OR (95% CI)	*P*-value
Length of hospital stay in days, mean (SD)	6.5 (5.8)	9.4 (7.4	− 0.169 (− 0.182, − 0.165)	< 0.001	− 0.158 (− 0.161, − 0.143)	< 0.001	− 0.162 (− 0.178, − 0.158)	< 0.001

Percentage given as percentage of column.

**Table 6:** Primary and secondary outcomes cTNM I–III—Linear regression analyses.

	Laparoscopic N = 4973	Open N = 7623	Unadjusted		Adjusted	
			OR (95% CI)	*P*-value	OR (95% CI)	*P*-value
Length of hospital stay in days, mean (SD)	6.5 (5.8)	9.4 (7.4)	− 0.172 (− 0.180, − 0.163)	< 0.001	− 0.149 (− 0.158, − 0.140)	< 0.001

Percentage given as percentage of column.

#### Pathological outcomes

R1 resections were significantly less frequent in the LAP group compared to the OPEN group despite adjusting for characteristics associated with a more advanced tumour including t-stage, n-stage, level of vascular- and perineural invasion and grade of cancer differentiation (1.8% vs 3.8%, unweighted regressions analysis OR 0.68, 95% CI 0.53, 0.89, *P* = 0.004 and weighted regressions analysis OR 0.68, 95% CI 0.58, 0.79, *P* < 0.001) (Table 3). R1 resections remained significantly less frequent in the LAP group compared to the OPEN group in the subgroup analyses including Stage I-III disease (Table 4). The proportion of resections containing less than 12 lymph nodes were not significantly different in the unweighted regression analysis LAP 5.8% vs OPEN 5.9%, OR 1.09, 95% CI 0.93, 1.28, *P* = 0.314 but this became significant in the weighted regressions analysis with OR 1.13, 95% CI 1.02, 1.25, *P* = 0.024 (Table 3).

## Discussion

This study demonstrated no difference in 30-day mortality following LAP or OPEN surgery in routine health care but in the weighted analysis there was a significantly lower mortality in the LAP group. There were also, significantly more re-operations within 30 days, re-admissions at 30- and 90 -days and longer hospital stays following OPEN surgery compared to LAP surgery in both the unweighted and the weighted analysis including the subgroup analysis. Also, there were more R1 resections in the OPEN group in both analysis methods and in the subgroup analysis for cTNM I-III. The overall 30- and 90- day mortality in this study is comparable to what has previously been reported in randomized trials but lower than what has been reported in other population based observational studies^1,14,15,26–28^.

Registry studies comparing short-term mortality following OPEN and LAP colon cancer resections have indicated a decreased mortality at 30- and 90- days for LAP surgery^7,13,15^. Few of these studies have adjusted for potential confounders including comorbidities and emergency surgery. Although this study included exclusively elective surgery there was a significant difference in 30- day or 90-day mortality. However, this only remained statistically significant in the weighted analysis for 90-day mortality following identification and adjustment for potential confounders, thus indicating a lack of causal relationship for 30-day mortality. The higher proportion of more comorbid patients in this study compared to randomized controlled trials is a true representation of the real-world colon cancer population in routine health care.

This study demonstrated a shorter hospital stay following LAP surgery which is in accordance with reports from previous randomized trials and registry studies^1,15,26^. Surgical morbidity including re-admissions has been reported to be higher following OPEN surgery in previous studies and reviews, however differences with regard to the rate of re-operation has so far, to our knowledge, not been reported^15,26,28,29^. The overall rate of re-operation within 30 days was slightly higher than what has generally been reported in the literature, despite similar rates of overall anastomotic leaks^26^. Comparing the two groups, a higher rate of re-operation was found in the OPEN group, which only in part could be explained by the slightly higher rate of anastomotic leak demonstrated in this group. Another potential explanation for this difference between the two techniques may be that re-operations due to less common complications such as wound dehiscence, small bowel obstruction and intraabdominal collections, may be more common following OPEN compared to LAP, and these differences may only be demonstrated in population based studies of sufficient size, such as this.

The overall re-admission rates observed at 30 and 90 days in this study are similar to previously reported rates from population studies^30^. Re-admission was significantly more common in the OPEN group at 30 and 90 days. The high proportion of comorbidities in this study compared to previous randomized trials may explain this, since LAP, when compared to OPEN has been found to decrease the risk of pneumonia, cardiac complications and wound infections, especially in the comorbid patients^31,32^. Cardiac, infectious and pulmonary complications are all common causes for re-admission, likely to be further pronounced in this more comorbid population^30,33,34^. It may be that comorbid patients have more to benefit from LAP, potentially explaining the difference in 30 and 90 day re-admission. This has previously been indicated by pooled study results^35,36^.

The proportion of R1 resections found in the OPEN group was higher than reported in randomized controlled trials but lower than proportions reported in population based studies^1,5,15^. The rate of R1 in the LAP group was similar to rates reported in randomised controlled trials. The overall increased rate of R1 resections in the OPEN group which remained significant despite adjustment for characteristics associated with more advanced tumours and in the weighted analysis is similar to findings reported in recent register studies^12,15^. R1 resection is a well-known negative prognostic factor affecting long-term survival and follow-up is required to evaluate if the increase in positive margins seen in this study will lead to long term survival benefits following LAP compared to OPEN as indicated in few population based studies^19,37^. Our unweighted analysis found similarly to previous registry studies no difference in the proportion of resections with 12 or more lymph node yield^5,18^. The significantly higher rate of resections with sufficient number of lymph nodes indicated in the weighted regression analysis has not been reported elsewhere. When interpreting these results it is worth knowing that a high poroportion of elective colonic resections in Sweden are performed by subspecialized colorectal surgeons, as many as 88% between 2007 and 2010, potentially explaining the encouraging outcomes in this study^6^.

Strengths of this study include the population-based setting and the combined use of two high quality registers, including nearly all patients diagnosed with colon cancer in Sweden 2012–2018 who subsequently underwent elective resection surgery. The combination of the two registries helped to minimize missing data and provided the means to accurately capture also 90-day re-admissions. High-quality detailed data from the SCRCR enabled getting access to information on potentially confounding variables like few other population-based studies previously have.

The limitations of this study are common to those of other register-based studies where selection bias and unmeasured confounding is present. Firstly, indications for choosing LAP or OPEN surgery, were not available. There was however, a predominance for more advanced cancers along with patients with more comorbidities in the OPEN group, indicating the presence of a selection process. Secondly, there is a potential risk of not sufficiently adjusting for confounding selection bias but the use of different statistical models demonstrating comparable results may have reduced the potential problem of residual confounding^38^. Thirdly, hospital and volume effects could not be accounted for.

In summary, this population-based study demonstrated no difference in 30- day mortality but an increased mortality at 90-days in the weighted analysis when comparing LAP with OPEN elective surgery in routine health care. The significantly lower number of re-operations and re-admissions in the laparoscopic group as well as the higher R0 resections indicate an advantage of laparoscopic surgery. Thus, considering previously published randomized trials and population-based studies establishing improved short-term outcomes and equal long-term outcomes, this study supports the standard use of LAP technique as first choice for colon cancer resections in routine health care.

## Data Availability

The datasets generated and analysed during the current study from the National Patient Registry and the Swedish Colorectal Cancer Registry under license from the current study and are not publicly available.
